# The effect of visually filled reproductions on the reproduced durations of auditory intervals

**DOI:** 10.3758/s13414-023-02755-9

**Published:** 2023-07-07

**Authors:** Miria N. Plastira, Marios N. Avraamides

**Affiliations:** 1 Department of Psychology & Center of Applied Neuroscience, University of Cyprus, P.O Box 20537, 1678 Nicosia, Cyprus; 2 CYENS Centre of Excellence, Nicosia, Cyprus

**Keywords:** Time perception, Time estimation, Reproduction, Auditory intervals

## Abstract

The present study examined how the perception of time is affected by the presence of a visual stimulus during the reproduction phase of an online time reproduction task. Participants were instructed to reproduce the durations of speed-altered speech snippets with either a picture or a blank screen presented during the reproduction phase. Results showed that fast speeches were reproduced as longer than slow ones, while the reproduced durations of short speeches were closer to the actual durations than were the long speeches. In addition, longer reproduced durations were observed in trials with a picture than in trials with a blank screen. These results provide clear evidence that postencoding information can influence the reproduction of previously encoded temporal intervals and are discussed in the context of attention allocation and its possible influence on an internal clock mechanism. Also, the study provides evidence that online testing is reliable for assessing biases in time perception, at least with time reproduction tasks.

The accurate perception of time is a crucial skill for our everyday life, implicated in a variety of tasks ranging from scheduling our daily program to keeping our social interactions as brief or as long as appropriate. Past research has documented that time perception is biased. That is, when judging how much time has passed or how long an event has lasted, people make systematic errors that are caused by stimulus properties and the context of the experience (e.g., Nather et al., 2011; Ono & Kawahara, 2007; Xuan et al., 2007). For example, when bored (Troutwine & O’Neal, 1981; Zakay, 2014) or when waiting for customer service (Antonides et al., 2002; Haynes, 1990), people judge time as passing more slowly. Also, when asked to reproduce, through a time reproduction task, the duration of intervals in which speech-like clips are presented auditorily, participants in studies are influenced by the speed of the speech, judging temporal intervals with fast speech as longer than equal intervals with slow speech (Plastira & Avraamides, 2020, 2021).

The time reproduction task is widely used in the time perception literature. It consists of the presentation phase in which a stimulus of a certain duration is presented, and the reproduction phase in which participants reproduce the previously presented duration, either by keeping a key pressed for equal time as the duration of the stimulus or by pressing a key once to indicate their reproduced duration. Past research on time perception that used this time reproduction task has suggested that the bias in time estimation may be due, at least partly, to attentional processes. In one study, Sgouramani and Vatakis (2014) had participants reproduce the duration of videos of a dancer performing dance steps in different speeds. After the presentation of each video, participants were presented with an image and were instructed to leave it on the screen for an equal time as the duration of the video. Results showed that participants perceived the duration of videos with fast steps as shorter than that of videos with slow steps. The authors explained this finding by arguing that videos of fast steps attracted attention towards the content of the video, and away from time-related processes, leading to underestimation of the video’s duration, while the opposite was the case with videos with slow steps (Sgouramani & Vatakis, 2014).

In line with this explanation, other studies have shown that time is indeed overestimated when attention is directed to the passage of time and underestimated when attention is focused elsewhere (S. W. Brown, 1997; Macar et al., 1994; Polti et al., 2018; Wearden, 2016; Zakay et al., 1983). For example, in a study by Zakay et al. (1983), participants were asked to reproduce the duration of intervals during which they performed verbal tasks of three levels of difficulty or nothing at all. In the easy task, participants read words that were written on cards, in the intermediate task they named objects presented on pictures, and in the difficult task they provided synonyms for words that were presented. Overall, results showed that the empty intervals were estimated as longer than those with a verbal task. Importantly, for intervals with verbal tasks, reproduced durations increased as the task became easier. Zakay et al. (1983) argued that this was the case because in easy tasks people focus their attention on the passage of time, while in difficult tasks they shift their attention more to the task. In line with this interpretation, a study of Polti et al. (2018) showed that the duration of a working memory task was judged as shorter compared with an empty interval of equal duration.

To account for the effects of attention on time estimation, Zakay and Block (1995) proposed the Attentional Gate Model. According to this model, while experiencing an event, the pacemaker of an internal clock generates pulses that pass through a gate into an accumulator (Block & Zakay, 2006; Zakay & Block, 1995). The rate with which the pulses are generated can be affected by the individual’s levels of arousal. Duration estimates are based on the number of accumulated pulses, with more pulses causing longer perceived durations. Notably, the model claims that, beyond the effects of arousal, attention may influence time estimation as well. That is, it posits that when attention is focused on time itself, the gate opens wider allowing more pulses to the accumulator (Fig. 1). In contrast, when attention is focused on nontemporal information such as the stimuli presented, the gate closes somewhat allowing fewer pulses to be accumulated (Wearden, 2016).

**Fig. 1:**
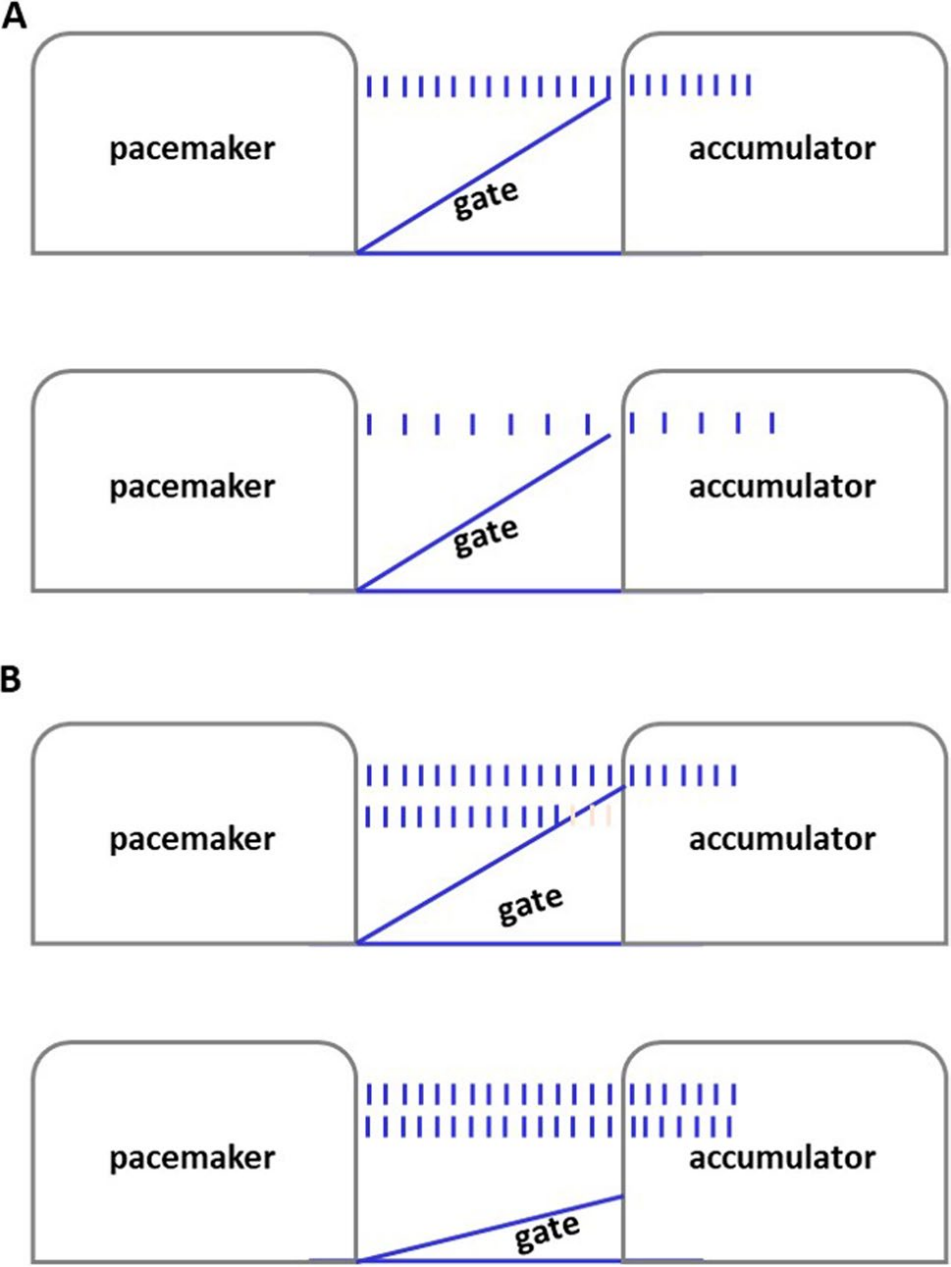
Schematic representation of the internal clock when (**A**) pulses are produced at different (pacemaker) rates and then pass from the gate to the accumulator, and (**B**) pulses are produced at a constant rate and pass from a narrow and a wide gate to the accumulator

Based on previous results (Van Volkinburg & Balsam, 2014) and on the assumptions of the Attentional Gate Model, originally proposed to account for effects during the encoding phase, we speculate that a stimulus presented during the reproduction phase of a task is also capable of diverting attention away from the timing process, narrowing the opening of the attentional gate, leading to the accumulation of fewer pulses at the unit of time. If this hypothesis holds true, people should overestimate the duration of a previously experienced event if a distracting stimulus is presented during the reproduction compared with when it is not. To our knowledge, the hypothesis that solely the presence of a stimulus, without explicit instructions to pay attention to it, can divert attention away from time and can thus influence the extend of reproduced durations, has not been tested before.

In the present study we carried out two experiments in which we examined whether the presence of a visual stimulus during the reproduction of the duration of an auditory stimulus, can bias the estimates of its duration. With the use of a time reproduction task, we evaluated participants’ ability to make prospective timing estimates. In the prospective paradigm approach participants know in advance that the temporal interval they experience will be estimated later. Following this approach, we explicitly instructed participants to track the passage of time without engaging in explicit counting (Gamache et al., 2011). More specifically, we asked participants to listen to speech-like audio clips with different durations and speeds and to reproduce their durations by pressing a key when they thought a period of time equal to the duration of the clip has elapsed from its presentation. In our previous work with a similar paradigm and stimuli but with no stimulus presented during the reproduction, we showed that participants overestimated the duration of fast stimuli (Plastira & Avraamides, 2020). In addition, speed influenced duration estimates so that faster stimuli led to longer reproduced durations, compared with slow ones.

Although no previous study has examined the effect of visually filled and empty reproductions on the reproduced durations, a number of studies have investigated whether the arousal/valence of nontemporal visual stimuli, presented during the reproduction phase, affects reproduced durations. For example, a study by Van Volkinburg and Balsam (2014) showed that the presentation of emotional visual stimuli associated with different levels of arousal, either during the presentation phase of a task or during the reproduction phase, affected the length of reproduced durations. Specifically, results showed that highly arousing (positive and negative) stimuli presented during the encoding of the intervals (presentation phase) led to longer reproduced durations, suggesting the speed-up of the pacemaker and the accumulation of more pulses during the encoding of the to-be-reproduced interval. The presentation of highly arousing stimuli during the reproduction phase led, again, to longer reproduced durations, but this result was attributed to the fact that highly arousing stimuli distracted attention from the timing process and led to the accumulation of pulses at a slower rate. In other words, more time was needed for the number of the previously experienced pulses to be accumulated, thus the reproduced duration was terminated later (Meck, 1996; Van Volkinburg & Balsam, 2014). Notably, this was found only for the highly arousing positive stimuli. Highly arousing negative stimuli presented during the reproduction phase led to shorter reproduced durations. Van Volkinburg and Balsam (2014) speculated that this was the case either because participants wanted to avoid seeing the negative images and terminated their presentation earlier, or that the arousing effect of these images distorted the pacemaker’s rate so heavily that the effect of attention allocation was overshadowed.

Furthermore, the results of other studies show that duration estimates are also affected by nonemotional characteristics, like stimulus complexity (Schiffman & Bobko, 1974), size (Rammsayer & Verner, 2015), subjectively perceived size (Ono & Kawahara, 2007), and numerical magnitude (Cai & Wang, 2014). Nevertheless, specific characteristics from those mentioned above, like the size of the stimuli (Rammsayer & Verner, 2014, 2015; Xuan et al., 2007) or the numerical magnitude (Cai & Wang, 2014), do not seem to have an effect on the reproduced durations, when presented during the reproduction of a previously experienced duration. More specifically, in the study of Rammsayer and Verner (2015), two experimental conditions were used. In one condition, a filled square or a filled circle, in one of two different sizes, was presented to participants for 800 ms, 1,000 ms, or 1,200 ms (target interval). After that, in the reproduction interval, a fixation cross appeared and participants had to leave it on the screen for as long as they thought that the stimulus presentation lasted, before pressing a button. The other condition followed the same procedure, but the fixation cross was presented during the target interval, while the nontemporal visual stimuli were presented during the reproduction interval. Results showed that while in the first condition (and for the 1,200-ms stimuli) the reproduced duration of the large visual stimuli was longer than that of the small stimuli, in the second condition, no effect of stimulus size on the reproduced duration was observed. The authors argued that if the size of the nontemporal visual stimuli modulates the function of the internal clock, then it would have had a significant effect on the reproduced durations in both conditions. Thus, they concluded that the size of the nontemporal visual stimuli does not affect the internal clock but the representation of time of the target interval that is stored in memory (Rammsayer & Verner, 2015).

Similarly, Cai and Wang (2014) suggested that the presentation of a nontemporal stimulus (i.e., a number), during a to-be-reproduced interval, affects the memory of the target duration. In their experiments, they presented intervals of four (300 ms, 450 ms, 600 ms, and 750 ms) different durations (perception stage) and asked participants to reproduce them by pressing a button for as long as they thought the intervals lasted. A small (1 or 2) or a large (8 or 9) number was presented on the screen, either during the perception stage (Experiment 1) or during the reproduction stage (Experiment 2). Results showed that, compared with small numbers, large numbers led to longer reproduced durations in Experiment 1; however, no effect of number magnitude on the reproduced duration was observed in Experiment 2. The authors concluded that the characteristics of these nontemporal visual stimuli influence the way the to-be-reproduced interval is stored in memory but not the functioning of the internal clock (Cai & Wang, 2014).

In summary, it seems that while the arousal induced by emotional visual stimuli during the reproduction phase affects the perception of time (Van Volkinburg & Balsam, 2014), other characteristics, such as their physical size and numerical magnitude, do not (Cai & Wang, 2014; Rammsayer & Verner, 2014, 2015; Van Volkinburg & Balsam, 2014; Xuan et al., 2007). If arousal biases duration estimates by engaging attention to the stimulus and away from the timing process, it is possible that this effect could be induced by simply presenting a neutral (i.e., nonarousing) stimulus during reproduction, under the assumption that any visual stimulus would somewhat capture attention. That is, it could be that a stimulus presented during a specific time frame, regardless of whether it is arousing or not, may capture attention, diverting the focus away from the timing processes and causing as a result the underestimation of its duration. Examining this hypothesis seems important for delineating the possible postencoding effects of attention on temporal estimations, independently of arousal. Unfortunately, none of the past studies has explored whether the mere presence of a neutral stimulus could influence duration estimates.

To address this gap in the literature and to examine the possible effect of attention during the reproduction phase, we compared here an empty reproduction condition with a filled reproduction condition, to examine whether a visual stimulus presented during the reproduction phase would (1) lead to overestimating the duration of normal speed stimuli and (2) yield an additive effect to that of speed by causing further overestimation of fast stimuli. To investigate both questions, we carried out two experiments in which we included both a manipulation of speed during in the presentation phase and a manipulation of the presence of a visual stimulus in the reproduction phase. A visual stimulus, rather than an auditory one, was used in the reproduction phase to prevent interference between the similar (potentially phonological) codes of maintaining the stimuli in memory in the two phases. In addition to the above aims, as the experiments were conducted online, they also served the purpose of replicating earlier results of our research conducted in the lab, providing thus a test for the reliability of online testing for conducting timing research. Notably, while some past studies in other fields (e.g., Hilbig, 2016; Segen et al., 2021) have replicated with online testing patterns of results obtained in the lab, other studies (e.g., Bochynska & Dillon, 2021) did not.

## Experiment 1

The experiment was carried out to examine whether the presence of a visual stimulus during the reproduction phase of a reproduction task would affect the reproduced duration. In a task carried out online, participants reproduced the duration of speech-like stimuli of different durations and speeds. During the reproduction phase, either a picture or an empty screen were presented, along with instructions. Based on the Attentional Gate Model (Zakay & Block, 1995), we expected the picture to bias attention away from the timing process causing an overestimation of the duration of the previously experienced audio clip. Based on the findings of Plastira and Avraamides (2020), we also expected that faster stimuli would yield longer reproduced durations than slow stimuli of the same duration. Of interest was to examine whether the potential effects of the speed of the stimulus and the presence of a picture at reproduction would be additive or not.

### Methods

#### Participants

Seventy participants (53 female) between the age of 16 and 58 years (*M* = 24.50, *SD* = 9.66) participated in this experiment. Participants were recruited by posts on social media and announcements in undergraduate and graduate psychology classes at the University of Cyprus. Electronic consent was obtained from all individuals that participated in the study. Before the online experiment began, participants were informed of the research purpose and were presented with the contact details of the researcher. They were also informed that their participation was voluntary and it could be terminated at any time. The experiment began only after a specific keypress that denoted that an individual agreed to participate in the study.

#### Stimuli

We created the time-reproduction task using lab.js, an open-source online experimental platform (Henninger et al., 2022). The task comprised a presentation phase and a reproduction phase. The stimuli presented in the presentation phase were speech-like audio clips that were used in our earlier study (Plastira & Avraamides, 2020). These stimuli were produced by accelerating and decelerating a speech audio track of a female musician reading a simple text in Greek. Before being accelerated or decelerated, the recorded audio track was cut to small segments of a few milliseconds that were then shuffled. The segments did not have a fixed duration, because we aimed to avoid interrupting a syllable and ending up with “unfinished” syllables in the final audio files. The resulting stimuli were combinations of four different durations (5 s, 6 s, 7 s, 8 s) and five different speeds (50%, 75%, 100%, 200%, 400%). Each combination of speed and duration was represented by two different audio clips, yielding a total of 40 different speech-like audio clips. Twenty of them were followed by an empty reproduction phase, and the rest were followed by a picture-present reproduction phase. The stimuli used in the picture-present reproduction phase were pictures of five male and five female cartoons depicting a head and the upper body. Each picture was presented twice. The pictures were downloaded from www.freepik.com.

#### Procedure

**Fig. 2:**
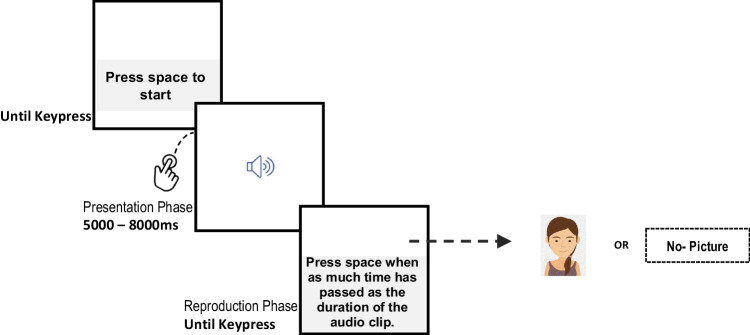
Schematic representation of a trial in the reproduction task of Experiment 1

Participants were instructed to carry out the task on a laptop or a desktop computer and wear headphones to listen to the sound. Upon clicking the URL of the online experiment, participants were asked to provide information regarding their age and gender. Τhe instructions of the task were then presented on the screen and participants were given unlimited time to read them before pressing a key to begin. Participants first carried out two practice trials before proceeding to the main experiment that included 40 trials, two trials for each combination of five speeds and four durations. The 40 audio clips were presented in a different random order for each participant. The structure of each trial is presented in Fig. 2. As seen in the figure, in the presentation phase of the trial an audio clip was presented to participants. During the clip, a speaker icon was presented on the screen. When the clip ended, participants had to reproduce its duration by pressing the space bar on the keyboard when they thought that that the time elapsed from the end of the clip was equal to its duration (reproduction phase). Participants’ key press was followed by a display asking them to press the space again for the next trial to begin (Fig. 2). The interval between the end of the sound and the space bar press, is referred to as the reproduced duration and represents the subjective estimation of the duration of the audio clip. In half of the trials, a picture was presented on the screen throughout the entire reproduction phase, while for the remaining trials the screen remained empty. In both cases, the instruction on how to respond was presented at the bottom of the screen.

### Results

A repeated-measures analysis of variance (ANOVA) was performed, with *speed*, *duration,* and *response condition* as the independent variables and *reproduction ratio* as the dependent variable. The response condition variable was a dichotomous variable that indicated whether a picture was present (picture-present condition) or absent (picture-absent condition) during the reproduction phase. The reproduction ratio was computed by dividing each reproduced duration by the actual duration of the presented speech audio file (reproduction ratio = reproduced duration/actual duration). A ratio greater than 1 indicates overreproduction, while a ratio smaller than 1 indicates under-reproduction (Mioni et al., 2014). A Greenhouse–Geisser correction (or the Huynh–Feldt correction when ε >.75) was applied when the sphericity assumption—tested with the Mauchly’s test—was violated.

Although the analysis revealed an overall underestimation of the duration of all stimuli, a larger reproduction ratio was found for the picture-present (*M* = .89, *SD* = .26) than the picture-absent condition (*M* = .85, *SD* = .25), *F*(1, 69) = 16.23, *p* < .001, η_p_^2^ = .19. In addition, results showed a main effect for speed, *F*(3.43, 236.48) = 15.14, *p* < .001, η_p_^2^ = .18. More specifically, the reproduction ratio increased with the speed. Within-subjects contrasts revealed that the reproduction ratio for the normal (100%) speed stimuli (*M* = .86, *SD* = .28) differed significantly from that of stimuli with 200% (*M* = .89, *SD* = .27), *F*(1, 69) = 4.46, *p* = .04, η_p_^2^ = .06, and 400% of the original speed (*M* = .94, *SD* = .27), *F*(1, 69) = 29.39, *p* < .001, η_p_^2^ = .30. Also, the reproduction ratio of the slowest (50%) stimuli (M = .83, SD = .24), differed significantly from that of stimuli with 200%, *F*(1, 69) = 10.55, *p* =.002, η_p_^2^ = .13, and 400% acceleration, *F*(1, 69) = 42.07, *p* < .001, η_p_^2^ = .38. Finally, the reproduction ratio of the fastest stimuli (400%), differed significantly from those of all the other stimuli (*p*s < .001).

**Fig. 3:**
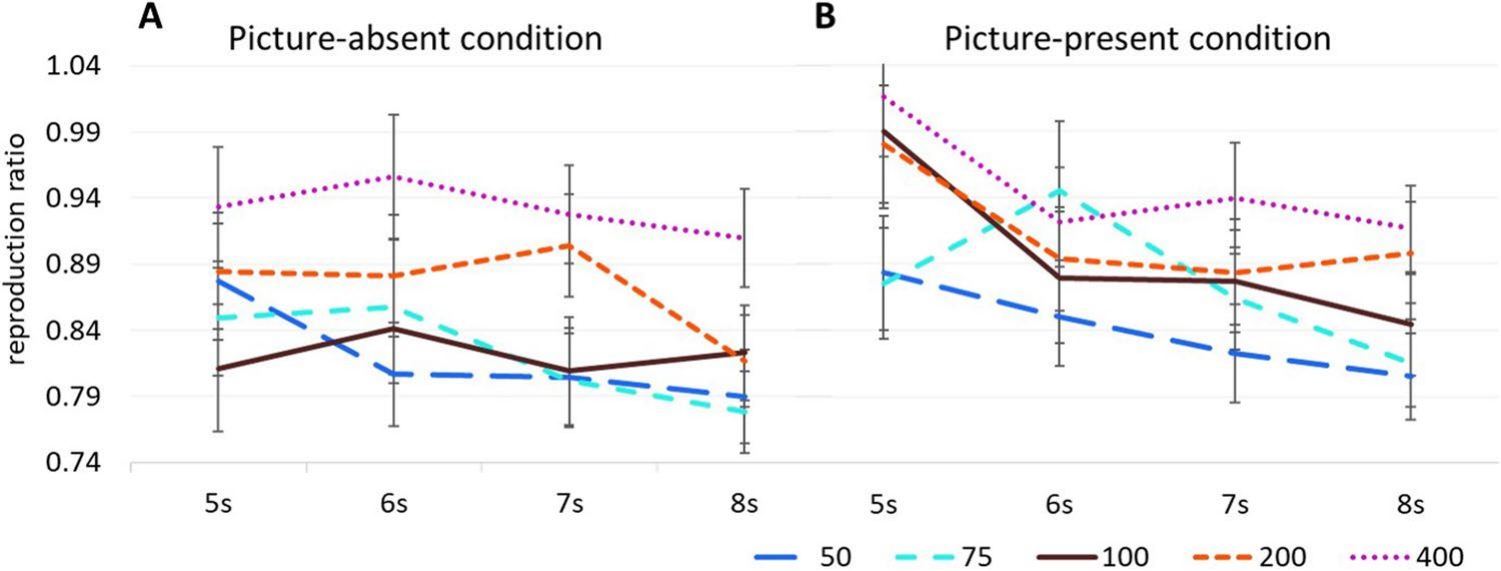
Reproduction ratio as a function of speed and duration for (**A**) the picture-absent condition and (**B**) the picture-present condition of Experiment 1. *Note.* Error bars represent standard errors. (Color figure online)

A significant main effect for duration was also obtained, *F*(2.06, 142.22) = 6.91, *p* = .001, η_p_^2^ = .09. Overall, the reproduction ratio decreased as the duration of the stimuli increased. Within-subjects contrasts showed significant differences between the reproduction ratio for the 5-s stimuli (*M* = .91, *SD* = .29), and the reproduction ratio of both the 7-s (*M* = .86, *SD* = .25) and the 8-s stimuli (*M* = .84, *SD* = .23), *F*(1, 69) = 7.06, *p* = .01, η_p_^2^ = .09, and *F*(1, 69) = 10.38, *p* = .002, η_p_^2^ = .13, respectively. The reproduction ratio of the 8s stimuli also differed significantly from that of the 6-s stimuli (*M* = .88, *SD* = .28), *F*(1, 69) = 8.17, *p* = .006, η_p_^2^ = .11. Notably, none of the interactions was significant (Fig. 3).

### Discussion

The results of Experiment 1 showed a larger reproduction ratio for the picture-present condition than for the picture-absent condition. That is, the speech stimuli were reproduced as longer when a picture of a cartoon human was presented during the reproduction phase of the task than when it was not, despite the fact that the picture was irrelevant to the task. This finding can be accounted for by the Attentional Gate Model (Zakay & Block, 1995). If the presence of the picture diverted attention away from the passage of time narrowing down the opening of the gate, fewer pulses would pass through to the accumulator. As a result, more time was needed in the picture-present than in the picture-absent condition, to accumulate the number of pulses that matched the duration of the previously presented audio clip.

Our results also revealed that as the duration of the speech stimuli increased, the reproduction ratio decreased. In other words, long durations were underestimated more than short durations. This finding follows Vierordt’s law (Brown, 1995; Lejeune & Wearden, 2009) and replicates the results of our previous study that used the same stimuli (Plastira & Avraamides, 2020), as well as the results of past research with visual stimuli (Brown, 1995; Sgouramani et al., 2019; Sgouramani & Vatakis, 2014).

Furthermore, the effect of speed observed here also replicates the results of our previous work carried out in the lab (Plastira & Avraamides, 2020), showing that the duration of fast speech stimuli is reproduced as longer than that of slow speech stimuli. Based on the internal clock model and the results of previous studies (Droit-Volet et al., 2013; Plastira & Avraamides, 2020), it seems that exposure to fast than slow auditory stimuli speeds up the rate of the internal pacemaker, leading to the accumulation of more pulses and to longer perceived durations. Importantly, as indicated by the lack of a significant Speed × Response Condition interaction, the effect of speed on temporal estimation was independent of the effect of the response condition, suggesting that biases caused by variables at and post encoding are distinct.

Notably, in contrast to our laboratory study (Plastira & Avraamides, 2020), we found no interaction between speed and duration. However, it should be mentioned that in the laboratory study we used a wider range of durations that included short speech stimuli of 2 s,3 s, and 4 s in addition to the 5-s, 6-s, 7-s, 8-s stimuli used here, and found that the effect of speed was greater for stimuli of short than long durations. It is therefore not surprising that we found no interaction in the current study that used only the longer durations.

Overall, our results show a clear influence of the presentation of an irrelevant visual stimulus at the time of reproduction, However, a question that arises is whether this was the case because the stimuli were pictures of humans, which are likely to be more attentional capturing than other kinds of pictures. Previous studies showed that a picture of a human face captures attention more than pictures of other objects (Langton et al., 2008; Ro et al., 2001). For example, in a visual search task involving the search of butterflies, the presentation of pictures of faces acted as distractor and affected the performance of the participants. Conversely, when butterflies were used as distractors, they did not interfere with the search for faces (Langton et al., 2008). To investigate whether our results are specific to the presentation of pictures of humans, in Experiment 2, we replaced them with pictures of other objects to examine whether their presence can cause the same distortions in the perception of time as the pictures of cartoon humans.

## Experiment 2

This experiment was conducted to examine whether the effect of a picture during the reproduction phase generalizes to pictures beyond those depicting human figures. Besides the difference in the nature of the irrelevant visual stimulus, Experiment 2 was identical to Experiment 1.

### Methods

#### Participants

Sixty-seven participants (49 female and 18 male) between the ages of 18 and 39 years (*M* = 21.40, *SD* = 3.48) participated in Experiment 2. Participants were recruited from psychology courses and were offered course credit for their participation. Participants were given the same instructions as those of Experiment 1 regarding the use of computer and headphones during the experiment.

#### Stimuli

The time reproduction task was identical to that of Experiment 1, except that the stimuli used in the presentation phase were pictures of 10 cartoon chairs. These pictures were also downloaded from www.freepik.com. Each picture was again presented twice through the experiment and was assigned to two specific stimuli of different speed–duration combinations, for all participants.

#### Procedure

The experimental procedure was identical to that of Experiment 1 (Fig. 2).

### Results

**Fig. 4:**
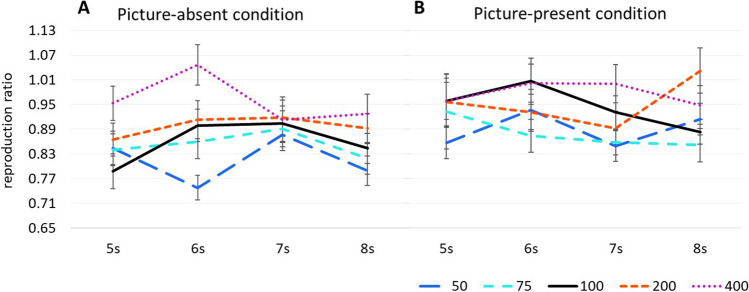
Reproduction ratio as a function of speed and duration for (**A**) the picture-absent condition and (**B**) the picture-present condition of Experiment 2. *Note.* Error bars represent standard errors. (Color figure online)

As in Experiment 1, we carried out a repeated-measures ANOVA, with speed, duration, and response condition as the independent variables and the reproduction ratio as the dependent variable. The analysis revealed main effects for the response condition and speed, *F*(1*,* 66) = 17.08, *p* < .001, η_p_^2^ = .21, and *F*(3.65, 241.090) = 12.99, *p* < .001, η_p_^2^ = .16, respectively. As in Experiment 1, the reproduction ratio was larger for the picture-present (*M* = .93, *SD* = .26) than for the picture-absent condition (*M* = .88, *SD* = .22). In addition, the results showed that as the speed increased, the reproduction ratio also increased. Within-subjects contrasts showed that the reproduction ratio for the normal speed stimuli (*M* = .90,* SD* = .25) differed significantly from those of all the other speeches (*p*s < .05) except from those with 200% (*M* = .92, *SD* = .25) of the actual speed (*p* = .20). In addition, the reproduction ratio for the slowest (50%) stimuli (*M* = .85, *SD* = .22) differed significantly from those of all the other stimuli (*p*s < .01) except from those at 75% (*M* = .87, *SD* = .26) of the actual speed (*p* =.40). Furthermore, the reproduction ratio for the fastest (400%) stimulus (*M* = .97, *SD* = .29) differed significantly from the stimuli at 75% (*p* < .001) and 200% (*p* =.02) of the actual speed (*M* = .92, *SD* = .25). In contrast to the findings of Experiment 1, the main effect for duration in Experiment 2 was not significant, *F*(2.605*,* 171.950) = 1.46, *p* = .23, η_p_^2^ = .02. This seems to be due to the 5-s (*M* = .90, *SD* = .26) stimuli which did not, as expected, yield a larger mean reproduction ratio than all the other stimuli. As expected, reproduction ratios decreased from 6-s (*M* = .92, *SD* = .25), to 7-s (*M* = .90, *SD* = .25), and then to 8-s stimuli (*M* = .89, *SD* = .24). As in Experiment 1, no significant interactions were observed (Fig. 4).

To compare the mean reproduction ratio across the two experiments, we carried out two independent-samples *t* tests. Results showed that the reproduction ratio for the picture-present condition was equal across Experiment 1 (*M* = .89, *SD* =.26) and Experiment 2 (*M* = .92, *SD* =.26), *t*(135) = −.78, *p* = .43. Similarly, for the picture absent condition, reproduction ratio for Experiment 1 (*M* = .85, *SD* =.25) was statistically equal to that of Experiment 2 (*M* = .88, *SD* =.22) experiment, *t*(135) = −.57, *p* = .57.

### Discussion

The results of Experiment 2 replicated the finding of Experiment 1 showing that the presence of an irrelevant visual stimulus during the reproduction phase increases reproduced durations compared with a control condition with no such stimulus. What is more, given that there was no difference in the reproduction ratio of the picture-present conditions across Experiments 1 and 2, results indicate that it makes no difference whether the irrelevant stimulus includes a human face or not.

Experiment 2 also replicated the effect of speed on time estimation of Experiment 1, showing that the fast speeches were perceived and reproduced as longer than the slow ones. As with Experiment 1, the effect of speed was additive to that of the response condition. That is, the presence of picture increased reproduced durations by the same amount of time in all different speeds. Regarding the effect of duration, results diverged a bit from those of Experiment 1. In Experiment 1, the reproduction ratio increased with duration. This pattern was observed in Experiment 2 as well, except for the shortest stimulus of 5 s, which did not yield the largest reproduction ratio as expected. It is not clear why this was the case.

## General discussion

The present study examined for the first time how the presence of a visual stimulus during the reproduction phase of a time reproduction task influences temporal estimations of auditory stimuli of various speeds. The auditory stimuli were also used in our prior work (Plastira & Avraamides, 2020) that examined the effect of speed and duration on the perception of time in a laboratory study. The present results replicated, for the most part, the effects of speed and duration of the past study while documenting a novel effect for the presence of a visual stimulus during the reproduction.

Specifically, results showed that accelerated speeches led to longer reproduced durations than slower and normal-speed speeches. These results are in line with those from previous studies suggesting that fast auditory stimuli speed up the internal pacemaker’s rate, leading to longer perceived durations (Droit-Volet et al., 2013; Plastira & Avraamides, 2020). In addition, results revealed that, in general, long stimuli are underestimated more than short ones. This result is in line with both Vierordt’s law (Brown, 1995; Lejeune & Wearden, 2009) and the findings of prior studies (Brown, 1995; Sgouramani et al., 2019; Sgouramani & Vatakis, 2014), including those from our own study that used similar stimuli (Plastira & Avraamides, 2020).

More central to the aims of the present study, results showed that the reproduced durations were longer in the picture-present compared with the picture-absent condition. In line with Zakay and Block (1995), our conjecture is that the pictures captured the attention of participants, diverting it away from the passage of time (Zakay & Block, 1995). We posit that by capturing attention, the pictures narrowed the opening of the internal clock’s attentional gate, allowing fewer pulses to pass to the accumulator during the reproduction phase. As a result, more time was needed for the accumulated pulses to reach the reference number of experienced pulses (i.e., the pulses accumulated during the presentation phase).

Notably, the effect of the response condition on reproduction duration was the same across the picture-present conditions of Experiment 1 and 2, which displayed a cartoon human and a cartoon chair, respectively. Therefore, across the two experiments, results indicate that a nontemporal visual stimulus presented during the reproduction of a duration can bias the reproduced duration, regardless of what it depicts. As reviewed in the introduction, Van Volkinburg and Balsam (2014) argued that arousing stimuli divert more attention away from the timing process during the reproduction causing an overestimation of previously encoded temporal intervals. Extending this explanation, we show here that, compared with an empty screen, even a neutral, nonarousing stimulus can cause over-reproduction, possibly by capturing attention away from the timing process.

In addition to the study of Van Volkinburg and Balsam (2014), a few other studies have presented visual stimuli during the reproduction phase of a task (Cai & Wang, 2014; Rammsayer & Verner, 2014, 2015; Xuan et al., 2007), but none of them has compared a filled reproduction to an empty one as we did. Results from these studies showed no effect of the extent to which the visual stimuli varied in size (Rammsayer & Verner, 2014, 2015; Xuan et al., 2007)or in numerical magnitude (Cai & Wang, 2014). Our results, on the other hand, indicate that, compared with an empty screen, a visual stimulus influences the timing process. A question that arises then is whether the influence of a visual stimulus during reproduction happens in an all-or-none manner. That is, it could be that a visual stimulus presented during reproduction biases time estimates but the extent of the bias remains the same, regardless of its features. Alternatively, it could be that the mere presence of a stimulus captures some attentional resources while further resources are directed to the stimulus depending on the bottom-up saliency of its features and/or how much its semantic content (that could elicit arousal) can influence top-down attention. This question could be tested by future research that can systematically manipulate the low-level properties and the semantic content of stimuli while at the same time including a control stimulus-absent condition.

Importantly, in the current study, the effect of response condition was additive to the effect of speed that was observed in both experiments. Indeed, for all speeds (and durations) the picture-present condition led to longer reproduced durations compared with the picture-absent condition. This suggests that different variables can exert distinct biases during the encoding of a temporal interval and during the retrieval. What is more, the mechanisms by which these biases occur might be different. For example, it could be that the pacemaker’s rate is more susceptible to influence during the encoding of an interval, while a stimulus is more likely to affect the function of the attentional gate during the retrieval of a duration. Here, we claim that fast auditory stimuli sped up the rate of the internal clock’s pacemaker while a picture during reproduction narrowed the opening of the accumulator gate. To shed more light on the mechanisms that influence time estimates, future studies may manipulate the same variables during encoding and reproduction (e.g., present the same visual stimulus during encoding and/or during reproduction).

Across the two experiments, our findings show that a stimulus from one modality (i.e., vision) presented during reproduction influences the duration estimates about a stimulus from another modality (i.e., audition). The effect of different modalities, mainly those of vision and audition, on the perception of time, has been examined in the past literature. Results from studies on the topic have shown that the durations of visual and auditory stimuli are experienced differently (Droit-Volet et al., 2007; Penney et al., 2000; Wearden et al., 1998, 2006), leading to a number of different interpretations and conclusions. For example, some researchers argue that a different pacemaker operates for each modality, with the auditory pacemaker running faster than the visual (Klink et al., 2011; Penney et al., 2000). Others argue that a single pacemaker is present but it runs at a different pace for each modality (Wearden et al., 1998). Furthermore, other evidence suggests a hierarchical structure for time perception, with a modality-specific mechanism that processes auditory and visual information separately and a modality-independent mechanism that processes information from both modalities (Stauffer et al., 2012). Although our results cannot differentiate these accounts, the cross-model influence we observed suggests that at least a unitary internal clock is present to process information from either modality. That said, it could be the case that some of its components (e.g., the pacemaker) are modality specific or are at least influenced differently by stimuli from different modalities. Our conjecture is that different components of the internal clock are affected by the stimulus characteristics during the presentation phase and the information presented during the reproduction phase (i.e., pacemaker vs. the attentional gate). Thus, to further understand the modality-specific influences of stimuli in these two phases, one may want to orthogonally manipulate the presence of visual and auditory stimuli in the two task phases.

Overall, our findings indicate that filled reproductions yield longer reproduced durations. Still, a study by Bratzke et al. (2017) derived the exact opposite conclusion. In Bratzke et al. (2017) participants were asked to listen to a continuous sine tone or to two sine tones with a gap and then reproduce the stimulus durations by either keeping a button pressed to indicate the duration or pressing the button twice to mark the beginning and the end of the reproduced duration. Results showed that keeping the button pressed, which could be regarded as a filled reproduction, led to shorter reproduced durations than pressing the button twice (Bratzke et al., 2017). Taken together with the findings of Bratzke et al. (2017), the current findings indicate that the presentation of a visual stimulus during reproduction affects time estimates differently from motor actions. It could be, for example, that whereas a visual stimulus captures attention away from the timing process, a sustained motor action attracts more attention towards it due to fatigue. Thus, our findings suggest that the modality or the type of stimulus/action that fills the reproduction interval may be critical for the direction of the biases in time estimation.

Finally, it should be noted that the results of the present study that used online testing, replicated for the most part those of our previous study that was conducted in the lab (Plastira & Avraamides, 2020), providing evidence that the online experiment platforms can be used effectively to conduct research on the perception of time. This was also supported by previous studies with adult samples that used different tasks, such as spatial memory tasks with static images (Segen et al., 2021), lexical decision tasks with reaction-time measurements (Hilbig, 2016), and preferential looking in children (Lo et al., 2021; but see Bochynska & Dillon, 2021, for divergent findings). Notably, past studies reveal greater variability in the online responses compared with the laboratory ones, suggesting thus that larger samples should be used for online studies (see Segen et al., 2021, for a discussion). The descriptive statistics form our study support this suggestion. In our online study, the standard deviation of the grand mean of the reproduction ratio was .25 for Experiment 1 (*n* = 70) and .24 (*n* = 67) for Experiment 2. Both values are a bit higher than the .19 of the laboratory experiment, obtained with a smaller sample (*n* = 40). These values seem to support the recommendation of Segen et al. that an online sample should be at least 25% larger than a laboratory sample to achieve similar variability.

To conclude, the present study provided clear evidence that the presentation of irrelevant, nonarousing visual stimulus during the reproduction of the duration of a temporal interval, can cause overreproduction. Importantly, this effect is independent of other biases caused by characteristics of the stimulus to-be-reproduced, such as its speed and actual duration. Our findings further our understanding about the factors that can bias time estimates. They also suggest that researchers must be cautious when comparing results across studies with differences in the methodological details of the reproduction tasks used, as even seemingly minor differences such as the presentation of an irrelevant visual stimulus during reproduction, can alter results.

## Data Availability

The datasets generated during and/or analysed during the current study are available from the corresponding author on reasonable request.
